# Primary Follicular Lymphoma of the Conjunctiva in a 12 Year-Old Male 

**Published:** 2013-04-22

**Authors:** Sh Taghipour Zahir, S A Miratashi, M Nazemian, S Zand

**Affiliations:** 1 Associate Professor of clinical and surgical pathology, Shahid Sadoughi university of Medical Sciences, Yazd, Iran.; 2 Assisstant Professor of ophthalmology, Shahid Sadoughi University of Medical Sciences, Yazd, Iran.; 3 Medical Student, Shahid Sadoughi University of Medical Sciences, Yazd, Iran.

**Keywords:** Lymphoma, Follicular, Conjunctiva

## Abstract

**Background:**

Follicular lymphoma (FL) is the second most common adnexal lymphoma of the eye that almost all of them are reported in elderly patients. Primary FL of the eye has been reported in only two children. Pediatric FL appears to be biologically distinct from typical adult FL. In cases without other organ involvement excision alone with close monitoring is a treatment of choiceand the prognosis is excellent.

**Case presentation:**

A 12 year -old male with a nodular lesion involving the inner can thus of the right eye was admitted to ophthalmology clinic. The lesion was painless and the nodule size gradually increased over a period of 6 months.Excisional biopsy demonstrated follicular lymphoma composed of neoplastic lymphoid cells which arranged in follicular pattern without germinal centers. Neoplastic cells were positive for Bcl2 and CD20.

**Conclusion:**

Although follicular lymphoma of the conjunctiva is rare in childhood but it could be considered as one of the differential diagnosis in this age group.

## Introduction

Extranodal lymphoma is rare, and about10% of them arise from ocular adnexa (1).Two types of non- Hodgkin lymphoma occurs in conjunctiva, one MALT lymphoma and the other is follicular lymphoma, and they are almost low grade B-cell type (2-4).Most ocular adnexallymphomas occurs in older age,and only a few cases have been reported in pedriatic previously (1-6).Herein, we report a case of primary follicular lymphoma of the conjunctiva in a twelve –year-old boy.

## Case presentation

A 12 year –old- male with a nodular lesion involving the right eye inner canthuswas admitted to ophthalmology clinic. The lesion was painless, and the size was increased over six months ago.There was no other ocular problem.His past medical history was normal.On physical examination there was no lymphadenopathy and hepatosplenomegaly.

**Figure 1:**
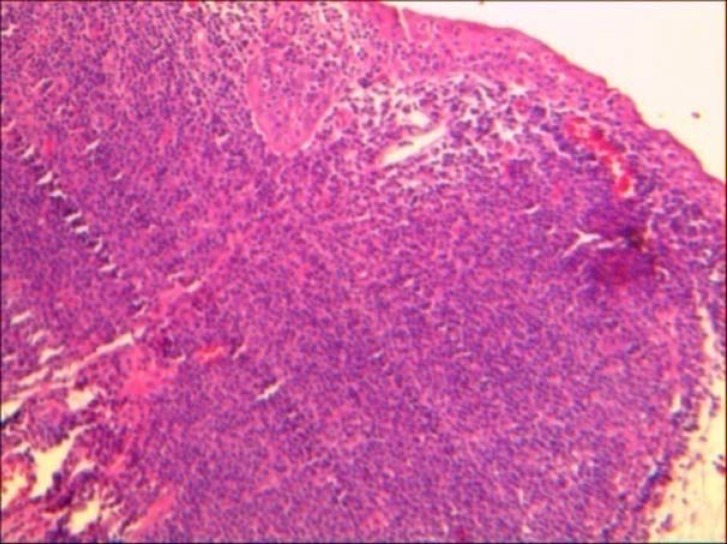
lymphoid cells infiltrated the conjunctival stroma. Conjunctival tissue involved by tumoral lesion composed of neoplastic lymphoid cells arranged in follicular pattern without germinal center X10

**Figure 2:**
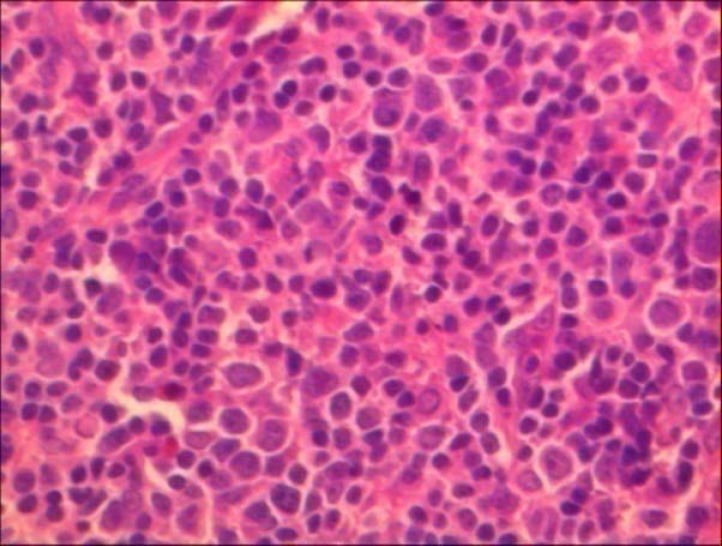
Follicular lymphoma composed of small cleaved and centrocyticlymphoid cells admixed with large cells.X20

**Figure 3:**
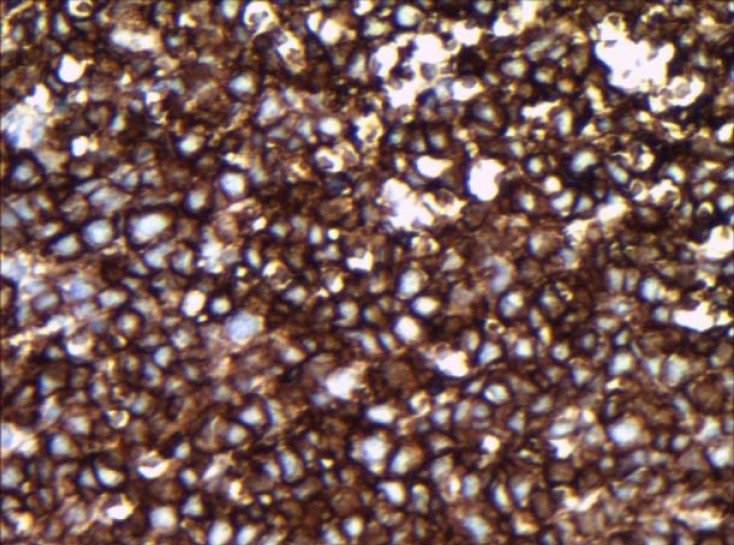
CD20 positive lymphoid cells

**Figure 4:**
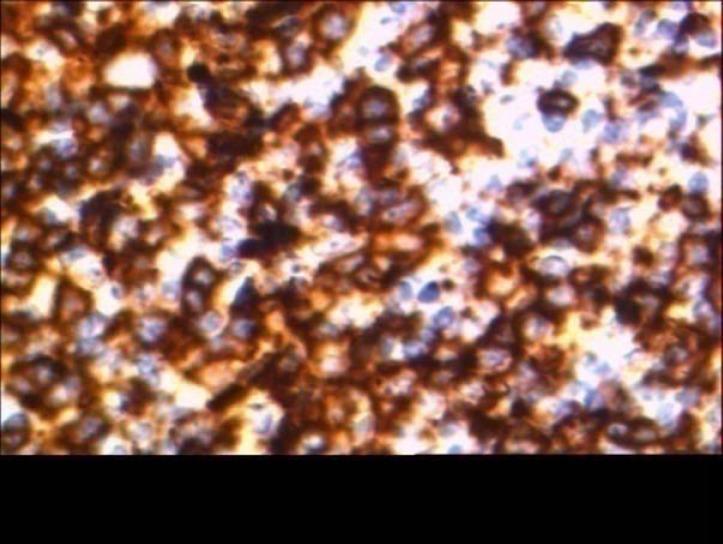
Lymphoid cells positive for Bcl2

Red blood cell and white blood cell counts with differential count were within normal limits.Ocular examination including visual acuityand indirect ophthalmoscopy were normal. He underwent an excisional biopsy of the lesion.Histopathologic examination revealed conjunctivalliningepithelium with the underlying stroma thatwas involved by lymphoid cells,and arranged in lymphoid follicle pattern without germinal center composed of small cleaved orcentrocytic lymphoid cells admixed with large cells (Figures 1,2). Mitotic figures wereconspicuous. Neoplastic cells filled the epithelium and stromal junction interface without invading the epithelium. All the surgical margins were free of tumor. For differentiation follicular lymphoma (FL) from reactive lymphoid hyperplasia immunophenotyping of lymphoid cells by immunohistochemical markers include ,bcl2,bcl6 ,CD20,CD45 and S-100 were studied and lymphoid cells were positive for CD20,bcl2 and bcl6 (Figures 3,4). So the diagnosis of FL was confirmed. In cytologic examination of bone marrow aspiration infiltration of neoplastic cells were not seen. Primary conjunctival follicular lymphoma without other organ involvementwas confirmed, and only monthly patient examination was recommended, and till now after nine months no recurrence was detected. Patient is in good health conditions.

## Discussion

Lymphoma is the most common orbital malignancy (3).Patients usually present with mass like lesion, pain, eye irritation, ptosis, excessive tear production or dropping eyelid (1-3).Most of them are localized at presentation and does not involve other organs. Most ocular lymphoma occurs in old age (5,6),and almost all of them are low grade B-cell lymphoma(2,4).FL is the second most common adnexal lymphoma (2), but almost all of them reported in elderly patients and only a few children with primary FL of the eye have been reported. One of them was a 6 year-old boy with 3mm mass in the conjunctiva which resected with clean surgical margin, and remained untreated. He was monitored closely and remained cancer free for 3 years till now (2).The other one was an 11 year- old girl with 25 mm mass involving the caruncle of the left eye underwent excisional biopsy and following the diagnosis, after staging studies there was no evidence of outside involvement, but based on unusual location of lesion, local radiation was administered over 28 days. She was cancer free till 2006 (7). FL is a member of lymphoma groups originated from germinal center-derived B cells. The main histopathological characteristics are recapitulation of lymph node follicles, lack of tangible body macrophages, crowded follicles of more than usual uniformity and lack of normal germinal centers (1-10). Lymphoid follicles in FL composed of small irregular cells both small cleaved cells or centrocytes admixed with large cells (both large cleaved and non- cleaved cells) (2-8). FL cells are positive for CD19,CD20, CD22, CD23 and CD10 in immunohistochemical studies. Rarely, FL arises in children or adolescents. A substantial proportion of these pediatric FL tumors appeared to be biologically distinct from typical adult FL. Characteristics of childhood FL include: Low stage disease (generally stage I/II), frequent involvement of the head and neck region, predominance of grade III histology, infrequent presence of bcl-2 protein expression (approximately 30 percent) or ofbcL-2 rearrangements (approximately 10 percent) and a high rate of apparent cure (9). The differential diagnosis of pediatric-type FL includes follicular hyperplasia and marginal zone lymphoma with prominent follicular colonization. Particularly follicular hyperplasia is much more common in young patients than FL. For differentiation of FL from reactive follicular hyperplasia, bcl2 and bcl6 are helpful markers, and FL cells are positive for these two markers (10). Although treatment of ocular follicular lymphoma has not been studied, but it appears that children with FL may do well following excision alone (without other treatments) and just close monitoring. The prognosis is excellent.We just follow up our patient every month,and until now (9 months after treatment) he remained tumor free.

## Conclusion

Primary follicular lymphoma of the conjunctiva is anextremelyrare tumor in children and should be differentiated with reactive lymphoid hyperplasia. For differentiation of these two lesions from each other, IHC has a significant rule.
